# Autopsy

**Published:** 1869-05-15

**Authors:** 


### COMMUNICATIONS.

BY N. SHERMAN, M.D.

PLYMOUTH, MARSHALL CO., IND., April 13, 1869.

CHICAGO MEDICAL JOURNAL:

By the politeness of Dr. J. D. Gray I witnessed the examtion of the body of Wm. Frantz, aged about twenty-five years. He was wounded in the U. S. service, in 1863, the ball entering at the lower portion of the ensiform cartilage and passing backwards and slightly upwards, making its exit on the same side just to the left of the spine. After the healing of the wound until April 5, 1869, his general health had been good. On that day, while making great exertion pulling at a rope, he was taken sick with severe pain in the stomach and vomiting. From that time until his death, eight days after, all medical aid gave no relief.

### *Autopsy.*

Twenty-four hours after death. Drs. Gray, Baily and Lessley. Considerable emaciation, abdomen sunken, left side of the chest much enlarged, after opening the chest and abdomen. The stomach was found where the left lung should be, much enlarged and distended with fluids and gas, compressing the left lung into a very small space at the superior portion of the lung cavity, at the same time forcing the heart wholly to the right side of the sternum, posterior to the ensiform cartilage, and to the left, in the line of the gun-shot wound was an opening from one and one-half to two inches in diameter, through the diaphragm. Through this opening the stomach proper, the large omentum and intestine were unmistakably strangulated, while the stomach except slight traces of inflammation, appeared quite healthy.

The thickened, smooth condition of the edges of the opening in the diaphragm clearly indicates that the opening had existed since the time of his wound. He had been, during the greater portion of the time for the past five years, actively engaged at hard labor, without suffering any pain or considerable inconvenience. That the passage of the stomach omentum and intestine through the opening in the diaphragm, compressing the lung, was recent, was evident from the fact that the lung was partially inflated to its normal condition.

